# Pituitary Insufficiency and Hyperprolactinemia Associated with Giant Intra- and Suprasellar Carotid Artery Aneurysm

**DOI:** 10.1155/2015/536191

**Published:** 2015-07-12

**Authors:** A. Gungor, N. Gokkaya, A. Bilen, H. Bilen, E. M. Akbas, Y. Karadeniz, S. Eren

**Affiliations:** ^1^Atatürk University Faculty of Medicine, Internal Medicine, Endocrinology and Metabolism Department, 25240 Erzurum, Turkey; ^2^Atatürk University Faculty of Medicine, Internal Medicine, 25240 Erzurum, Turkey; ^3^Erzincan University Faculty of Medicine, Internal Medicine, Endocrinology and Metabolism Department, 24000 Erzincan, Turkey; ^4^Atatürk University Faculty of Medicine, Radiology Department, 25240 Erzurum, Turkey

## Abstract

Pituitary insufficiency secondary to internal carotid artery (ICA) aneurysm is a very rare condition. Its prevalence is reported as 0.17% (Heshmati et al., 2001). We present a case of pituitary insufficiency and hyperprolactinemia secondary to suprasellar giant intracranial aneurysm. A 71-year-old man was admitted to our clinic with symptoms of hypopituitarism, hyperprolactinemia, and visual field defect. His pituitary MRI and cerebral angiography revealed a giant saccular aneurysm filling suprasellar cistern arising from the ophthalmic segment of the right ICA. Endovascular treatment was performed on the patient to decrease the mass effect of aneurysm and improve the hypophysis dysfunction. After treatment, his one-year follow-up showed the persistence of hypophysis insufficiency, decrease of prolactin (PRL) level, and normal visual field. An intracranial aneurysm can mimic the appearance and behavior of a pituitary adenoma. Intracranial aneurysms should be taken into consideration in the situation of hypopituitarism and hyperprolactinemia. It is important to distinguish them because their treatment approach is different from the others.

## 1. Introduction

Annual incidence of hypopituitarism is 4.2/100000 cases. The most common etiology of hypopituitarism is the mass effect of pituitary and nonpituitary tumors. Pituitary adenomas, nonpituitary tumors like craniopharyngiomas, meningiomas, gliomas, chordomas, metastases, and cerebral aneurysms cause extrinsic compression of the hypothalamus, pituitary gland, and stalk resulting in pituitary insufficiency [1]. Also pituitary stalk compression may lead to hyperprolactinemia because of withdrawal of the dopaminergic inhibitory control. Cerebral aneurysms are a very rare reason for hypopituitarism that may be responsible for less than 0.2% of cases. There are only 40 cases in the literature up to this time [2]. We present here a case of a giant ICA aneurysm with findings and symptoms of pituitary insufficiency and hyperprolactinemia.

## 2. Case Report

**Figure 1:**
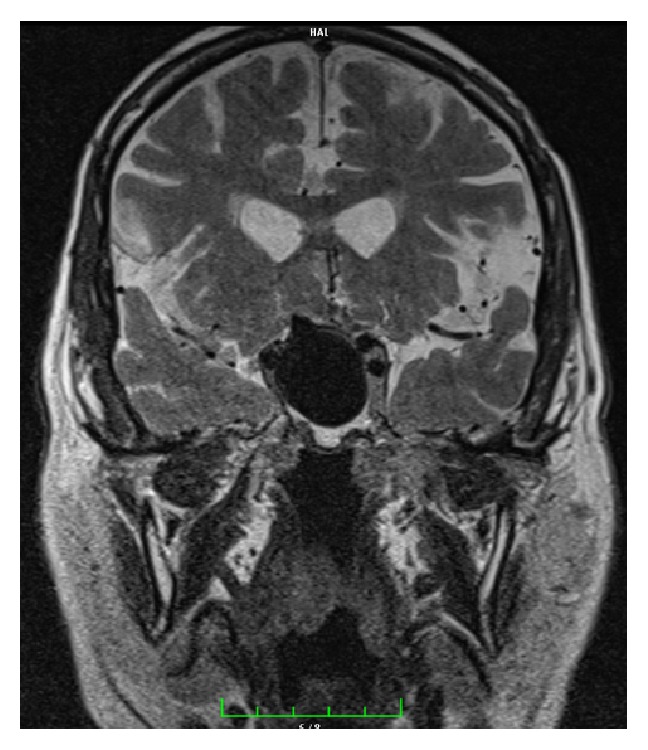
Coronal T2-weighted MR image showing giant saccular aneurysm.

**Figure 2:**
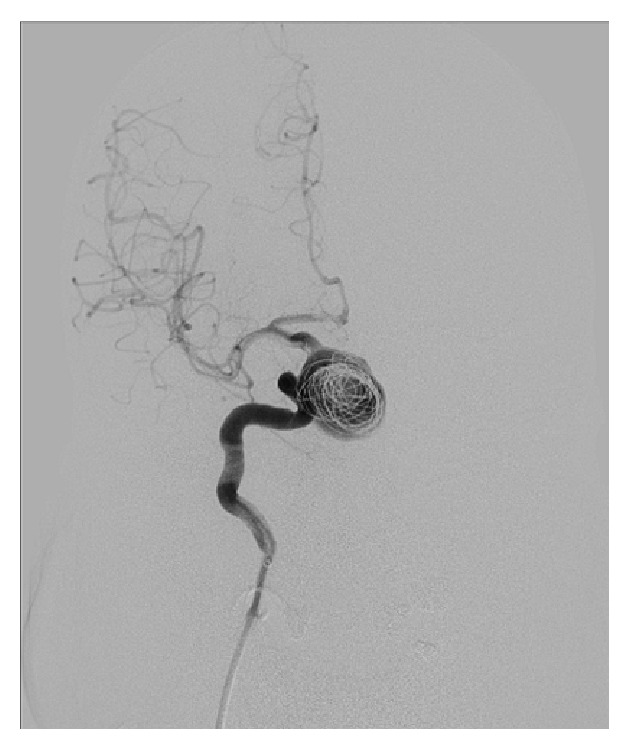
Angiographic image of endovascular treatment with flow-diverter stent and coils.

**Figure 3:**
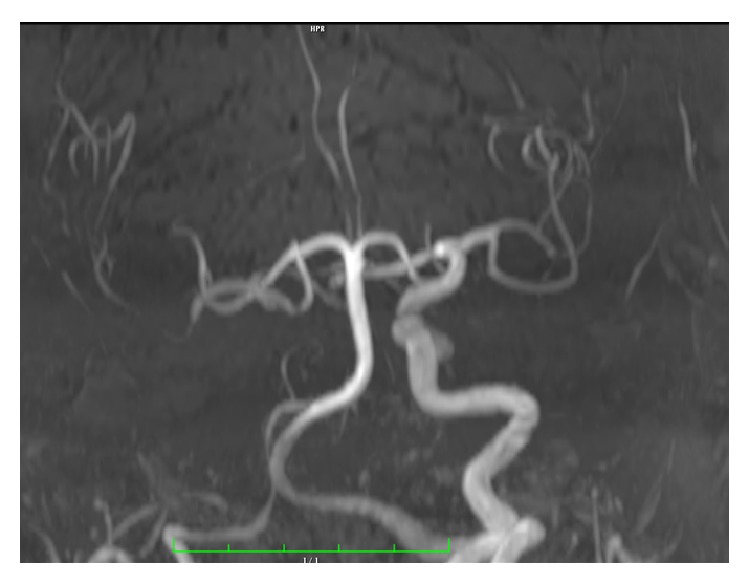
MR angiography image after embolization.

**Figure 4:**
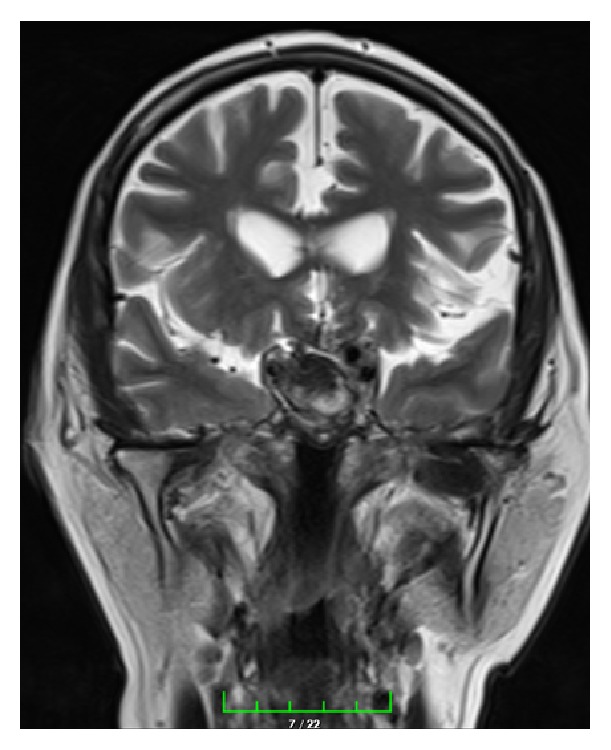
Coronal T2-weighted MR image after embolization.

A 71-year-old male patient was referred to our clinic with lassitude, fatigue, weakness, nausea, and headache. Except systemic hypertension there was no significant disease, in his medical history. In physical exam arterial blood pressure was 90/70 mmHg, pulse rate was 67/min, and he had no fever. Other systems were normal except that ophthalmological examination revealed a visual field deficit. Results of laboratory tests were as follows: GH: 0,105 ng/mL (N: E < 1), IGF-1: 45 ng/mL (according to age and gender normal range 64–188), 64 FSH: 0,18 mIU/mL (N: 1,27–19,26), LH: 0,1 mIU/mL (N: 1,24–8,62), total testosterone: 0.9 ng/mL (>18 age male normal range: 1.75–7.81), free testosterone: 0.6 ng/mL (>18 age male normal range: 1,10–3,10 ng/mL), ACTH: 3,68 pg/mL (N: 0–46), prolactin: >211 *μ*g/L (N: 2,4–13,3), TSH: 3,38 *μ*IU/mL (N: 0,34–5,60), FT4: 0,28 ng/dL (N: 0,61–1,12), fasting cortisol: 1,95 *μ*g/dL (N: 6,7–22,6), Na: 134 mmol/L (N: 135–145), urinary density: 1013, and spot urinary osmolarity: 200 mosm/mL. Pituitary MRI was taken of the patient with a preliminary diagnosis of pituitary insufficiency. Interpretation of pituitary MRI imaging was as follows: right ICA, approximately 25 × 29 mm size lesion resembling giant saccular aneurysm having continuity with C5 segment and filling suprasellar cistern (Figure 1). High level of prolactin and current pituitary insufficiency were considered as secondary to mass effect of suprasellar carotid artery aneurysm. As a result of low level of LH and total and free testosterone hypogonadotropic hypogonadism, normal TSH and low FT4 secondary hypothyroidism, low level of ACTH, and fasting cortisol central adrenal insufficiency diagnosis were thought. Central diabetes insipidus was not thought because of urine density and spot urine osmolarity was in normal range and polyuria was absent. For secondary hypothyroidism and central adrenal insufficiency, levothyroxine 75 mcg/day and hydrocortisone 20 mg/day were started. Because of social-cultural and social-economic reasons growth hormone and gonadotropin therapy was not prescribed to the patient. To prevent aneurysm rupture and decrease mass effect, embolization of right ICA aneurysm was performed with flow-diverter stent and coils (Figure 2). Following one month after intervention, control MR angiography revealed no flow at right ICA due to ineffective use of medications (Figure 3) and control MR imaging showed decrease of aneurism sac size with total thrombolization (Figure 4). Right MCA and ACA showed cross filling with anterior communicant artery while there was no side sign. His one-year follow-up showed the persistence of hypophysis insufficiency, decrease of PRL level, and normal visual field.

## 3. Discussion

Sellar-suprasellar aneurysm associated with pituitary insufficiency is a very rare entity. In only 7 of 4097 patients with pituitary insufficiency (0, 17%) the etiology was found to be intrasellar aneurysm in a study carried out by Heshmati et al. [3]. Although ICA aneurysms, particularly those located at sellar-suprasellar region, are very rare, they pose difficulty in differential diagnosis since they resemble pituitary tumors in terms of imaging and laboratory findings. Even though the mechanism by which endocrine dysfunction occurs in ICA aneurysm located at sellar-suprasellar region is not clear, two mechanisms are considered as liable. First, as a result of aneurysm's compression on hypothalamus of pituitary gland pedicle, effects of pituitary activating and inhibiting factors released from hypothalamus on pituitary gland are disrupted. The second one is destructive effect of enlarging aneurysm on pituitary gland [4]. Imaging methods as well as prolactin and other pituitary hormone measurements are valuable tools in differential diagnosis of aneurysm and pituitary tumors in mass lesions detected at sellar-suprasellar region. In our case, prolactin levels were considerably high and other pituitary hormone levels were low. Either identified mass is itself a prolactin releasing pituitary tumor or this mass leads to prolactin rise by preventing secretion of prolactin inhibiting factor (dopamine) by compression on hypothalamus or pituitary stalk. If the latter is considered more likely as etiology of high prolactin level, then conditions like craniopharyngioma, meningioma, dysgerminoma, tumors arising from the third ventricle space, nonfunctional pituitary tumors, infiltrative diseases such as sarcoidosis and eosinophilic granuloma, empty-sella syndrome, and lymphocytic hypophysitis in addition to sellar-suprasellar ICA aneurysm should also be excluded [5]. Before modern imaging techniques aneurysms were misidentified as pituitary tumors, but now with new techniques it is possible to detect them. Higher prolactin levels usually suggest the presence of a pituitary tumor (prolactinoma) [5]. Stalk effect which occurs with reduction of dopaminergic inhibition typically presents with prolactin levels of 30–200 ng/mL [6]. In our patient, because of levels of PRL higher than 200 ng/mL and MR imaging method showed absence of sellar-suprasellar mass, prolactinoma diagnosis was excluded. Also MR angiography and interventional cerebral angiography were performed and they presented that lesion was an aneurysm. After the treatment of aneurysm with embolization method his PRL levels decreased. These findings led us to excluding prolactinoma diagnosis. Taking decision relying on prolactin levels is more difficult; however, our suspicion of any other sellar or suprasellar mass lesion and carrying out imaging studies in this direction helped us reach the exact diagnosis. Our case emphasizes that a very high level of PRL can be as a result of stalk effect and nonpituitary masses should be kept in mind for differential diagnosis.

Literature search has shown that as well as higher prolactin levels in most of the patients with sellar and suprasellar aneurysm there was hypogonadism in 67.5%, adrenal insufficiency in 48.6%, and hypothyroidism in 40.5% [7]. In our case, hyperprolactinemia, hypogonadism, hypothyroidism, and adrenal insufficiency were present and lack of growth hormone was identified in addition to all these hormonal abnormalities.

Treatment of large sellar or suprasellar aneurysm is considerably difficult. The primary goal of treatment is to prevent aneurysm rupture. Microsurgical procedures, invasive endovascular interventions for obliteration of aneurysm, and hormone replacement therapy are used in treatment of this condition. In this patient group, close follow-up is necessary due to the fact that inadequate occlusion with endovascular interventions carries a future risk of growth and rupture [8].
